# Integrative study of pulmonary microbiome, transcriptome and clinical outcomes in *Mycoplasma pneumoniae* pneumonia

**DOI:** 10.1186/s12931-024-02687-4

**Published:** 2024-01-18

**Authors:** Xia Huang, Yingying Luo, Jing Wang, Xuefang Zhang, Lei Chen, Ruxi Wu, Zhengyang Xue, Haiyan Gu, Daiying Li, Heng Tang, Houbing Qin, Deyu Zhao, Feng Liu

**Affiliations:** 1 Department of Respiratory Medicine, Children’s Hospital of Nanjing Medical University, Nanjing, 210008 China; 2 Vision Medicals Center for Infectious Diseases, Guangzhou, 510705 China

**Keywords:** Host response, Microbiome, *Mycoplasma pneumoniae*, Neutrophils, Outcomes

## Abstract

**Background:**

This study aimed to investigate the interactions among three core elements of respiratory infection—pathogen, lung microbiome, and host response—and their avocation with the severity and outcomes of *Mycoplasma pneumoniae* pneumonia (MPP) in children.

**Methods:**

We prospectively collected bronchoalveolar lavage fluid from a cohort of 41 children with MPP, including general MPP (GMPP) and complicated MPP (CMPP), followed by microbiome and transcriptomic analyses to characterize the association among pathogen, lung microbiome, and host response and correlate it with the clinical features and outcomes.

**Results:**

The lung microbiome of patients with CMPP had an increased relative abundance of *Mycoplasma pneumoniae* (MP) and reduced alpha diversity, with 76 differentially expressed species. Host gene analysis revealed a key module associated with neutrophil function and several inflammatory response pathways. Patients with a high relative abundance of MP, manifested by a specific lung microbiome and host response type, were more prone to CMPP and had a long imaging recovery time.

**Conclusion:**

Patients with CMPP have a more disrupted lung microbiome than those with GMPP. MP, lung microbiome, and host response interacts with each other and are closely related to disease severity and outcomes in children with MPP.

**Graphical Abstract:**

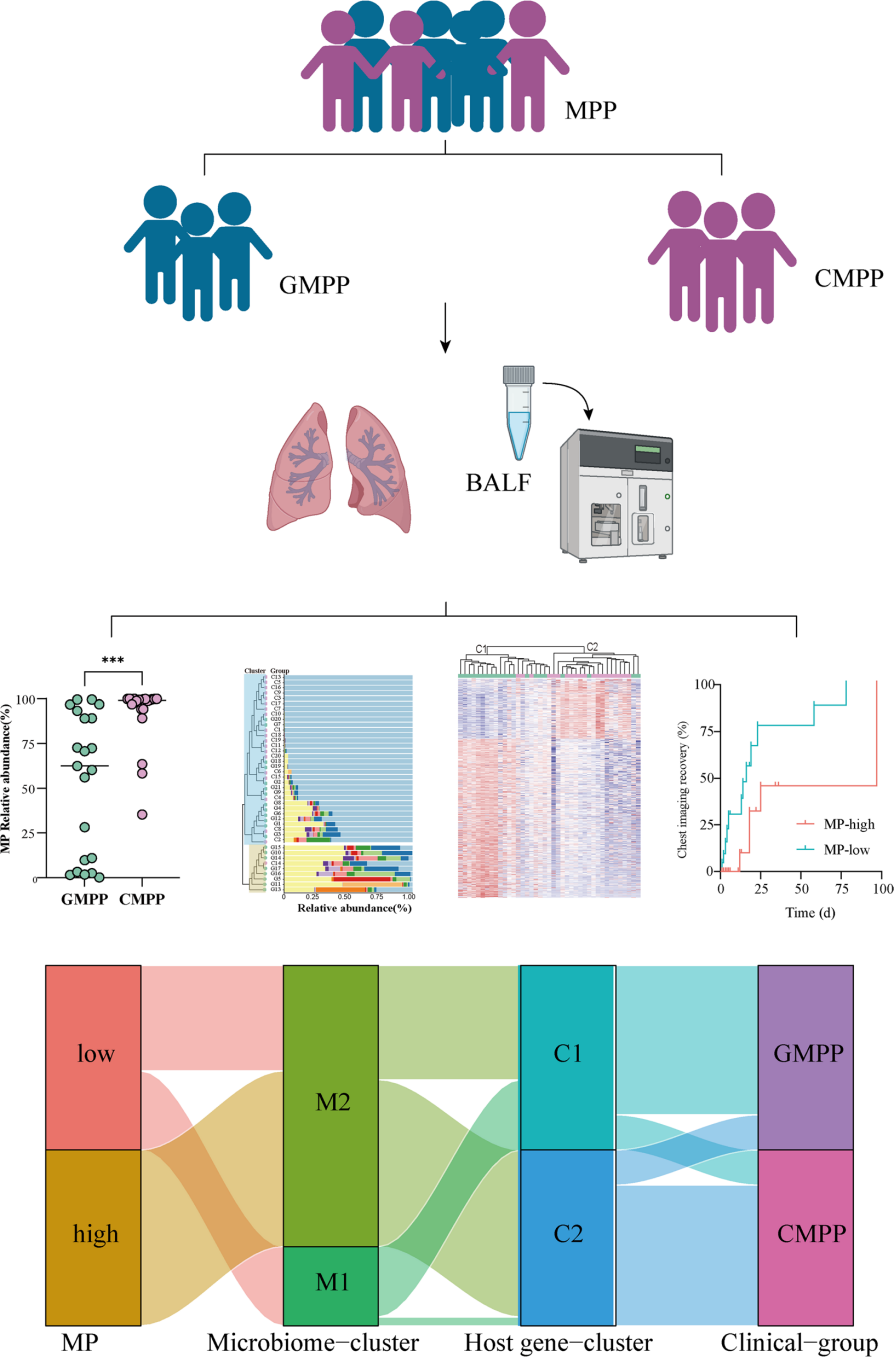

**Supplementary Information:**

The online version contains supplementary material available at 10.1186/s12931-024-02687-4.

## Introduction

*Mycoplasma pneumoniae* (MP) is one of the main pathogens of community-acquired pneumonia (CAP) in children. Clinical manifestations of Mycoplasma pneumoniae pneumonia (MPP) vary and can be accompanied by multiple complications, ranging from local complications such as parapneumonic effusion [1], and necrotizing pneumonia [2], to systemic complications such as respiratory failure [3], which could be defined as complicated CAP (CCAP) [4]. However, the exact mechanism of CCAP progression in patients with *M. pneumoniae* pneumonia (MPP) requires further investigation.

Host responses are essential in MPP development. Patients with CCAP in MPP are often characterized by persistent fever and high levels of inflammatory indicators, implying a strong systemic inflammatory host response [1, 5]. Sequencing of MP-infected epithelial cells indicated MP’s inherent ability to modulate host immune pathways [6]. MP infection-mediated lung injury was significantly mitigated in immunosuppressed hosts [7]. Additionally, the improvement of clinical symptoms with proper treatment using glucocorticoids and immunoglobulins suggests a key role of host response in MPP [8].

The respiratory microbiome also contributes to pulmonary immune response regulation [9]. Compositional and functional changes in this microbiome disrupt the overall host-microbiota balance, altering mechanisms of colonization resistance, which in turn affect infection outcomes [10]. Multiple studies have associated an altered airway microbiome with respiratory diseases, including ventilator-associated pneumonia, asthma, and bronchiectasis [11–13]. The airway microbiome in patients with MPP is imbalanced and related to disease severity [9, 14, 15]. Pathogens, lung microbiome, and host response are the three core elements of respiratory infections [2]. However, the correlation among MP, airway microbiome, and host response in MPP remains poorly understood.

We performed metagenomic next-generation sequencing (mNGS) and transcriptomic analysis of the bronchoalveolar lavage fluid (BALF) from a cohort study of patients with MPP. One of our previous studies revealed that the lung microbiota in the BALF was not significantly different between the severe and opposite sides [16]; however, we did not group the children according to the disease severity. In the present study, we investigated the interactions among the three core elements of respiratory infection and their association with disease severity and outcome by analyzing the sequencing results for a better understanding of MPP development and progression.

## Methods

### Patients and clinical information

Our study included patients with MPP hospitalized in the Department of Respiratory Medicine at the Children’s Hospital of Nanjing Medical University between January and December 2021. This study was approved by the research ethics committee of our institution (approval number: 202012089-1) and complied with the Declaration of Helsinki. The parents of all participating children provided informed consent before their inclusion in the study.

Inclusion criteria: (1) presence of fever and respiratory symptoms; (2) pneumonia confirmed by chest X-ray; and (3) positive serologic test (positive serum MP Immunoglobulin M, or seroconversion in paired sera) and positive MP polymerase chain reaction (DNA expansion increments ≥ 1 × 10^3^ copies/mL) results for nasopharyngeal aspirates.

Exclusion criteria: patients (1) with bronchopulmonary dysplasia, congenital heart disease, immunodeficiency, or hereditary neurological disorders, (2) coinfected with other pathogens, as determined by virus testing, nasopharyngeal aspirate culture, blood, alveolar lavage fluid, or pleural effusion culture, and (3) who did not agree to participate in this clinical study.

Persistent fever or poor chest imaging performance following adequate care and treatment with macrolide antibiotics indicated the need for Bronchoscopy, which was performed under intravenous-inhalation combination anesthesia. BALF samples were collected and stored at − 80℃.

Complicated MPP (CMPP) was defined as the presence of a combination of local (e.g., parapneumonic effusion, empyema, necrotizing pneumonia, and lung abscess) and systemic (e.g., bacteremia, metastatic infection, multi-organ failure, acute respiratory distress syndrome, disseminated intravascular coagulation, and rarely death) complications [4]. Otherwise, MPP was general MPP (GMPP).

Information was prospectively collected from patients’ medical records. The clinical information included age, sex, extrapulmonary complications, total fever duration, length of hospital stay, and hospitalization expenses. Laboratory data included white blood cell (WBC) count, neutrophil percentage (N%), C-reactive protein (CRP), alanine transaminase (ALT), aspartate transaminase (AST), lactate dehydrogenase (LDH), and D-dimer levels.

Nucleic acid extraction of BALF samples, library preparation, and sequencing were described in our previous study [16].

### Lung microbiome analysis

Microbial reads were obtained by mapping the ID-seq database sequence data with host sequences removed using SNAP v1.0 beta.18. The primary abundance compositions of the microorganisms in the CMPP and GMPP groups at the species level were visualized. Total bacterial load, MP load, MP relative abundance were quantitative skewness data, presented as median (interquartile range), and were analyzed using the Wilcoxon–Mann–Whitney rank-sum test. Percent of MP as the most dominant was categorical data are expressed as frequencies and examined using the chi-square test.

The vegan package in R software was used to analyze the microbiome’s alpha and beta diversity of the microbiome. Alpha diversity metrics (ACE, Chao1 estimator, Shannon and Simpson Indices) presented as median (interquartile range), and were analyzed using the Wilcoxon–Mann–Whitney rank-sum test. Beta diversity was measured by the Bray-Curtis distance test. Principal coordinate analysis (PCoA) and nonmetric multidimensional scaling (NMDS) were used to visualize similar distances between samples. Relative abundance differences of the microorganisms ranked in the top 10 between the GMPP and CMPP groups at the species level were displayed. The samples were clustered based on the relative abundance of species in each sample and the Bray-Curtis distance.

The microbiome network was constructed using the CoNet plug-in in Cytoscape. Before constructing the networks, we removed all taxon in the sample that were less than the minimum occurrence value of 20 and converted the count to the relative abundance. Permutations and bootstraps were performed 100 times each, and then the 2 distributions were compared using a z-test for each of the 4 methods including 2 measures of correlation (Pearson and Spearman) and 2 measures of dissimilarity (Bray-Curtis dissimilarity and Kullback-Leibler divergence) [17]. The associations between bacterial genera from each of the 4 methods were ranked by correlation coefficients or dissimilarity values and prefiltered so that, for each measure, the top 1000 and bottom 1000 of associations were identified for input into the downstream network reconstruction algorithm. The detailed procedure was referred to the previous studies [17, 18]. Cytoscape 3.9.1 was used for network visualization.

The differentially expressed species (DES) of the microbiome ranked in the top 10 between the CMPP and GMPP groups were selected using linear discriminant analysis (LDA) effect size (LEfSe). The DESs were determined by an LDA score > 2.0, and a *P* < 0.05. The microbiomeMarker R package was used for LEfSe.

### Transcriptomic evaluation

Read count normalization and differential expression analyses were performed using the DESeq2 package. Differentially expressed genes (DEGs) were determined with an adjusted *P* < 0.05 and an absolute Log_2_FC > 1. The clusterProfiler package was used for Gene Ontology (GO) and Kyoto Encyclopedia of Genes and Genomes (KEGG) pathway enrichment analyses. Benjamini-Hochberg adjusted *P* < 0.05 showed significant enrichment.

### Exploring immune cell infiltration

To infer the composition of immune cells, the CIBERSORT algorithm with the original CIBERSORT gene signature file LM22 and 1,000 permutations was used to examine the relative proportions of the 22 invasive immune cell types in each sample [19]. We also validated neutrophil infiltration using two other algorithms (Single-sample Gene Set Enrichment Analysis (ssGSEA) and MCPcounter. All the data were presented as median (interquartile range), and were analyzed using the Wilcoxon–Mann–Whitney rank-sum test.

### Weighted gene co-expression network analysis (WGCNA)

Based on the gene expression profiling, the goodSamplesGenes method in WGCNA package of R software was used to remove the outliers of genes and samples [20]. Then the MAD (Medium Absolute Deviation) of each gene was calculated to retain the top 50% of the genes. The Euclidean distance between the samples was calculated to remove an abnormal sample by clustering. WGCNA was carried out by the WGCNA R package [20], with the following parameters: β = 10, minModuleSize = 100, and mergeCutHeight = 0.4. The correlation coefficients and corresponding *P*-values between the different modules and clinical traits were calculated and visualized using a heat map. The module showing the highest correlation with the clinical features was identified as the key module. The intersection of key genes in the key module and DEGs was selected to explore the module biological function through GO and KEGG enrichment analysis.

### Protein–protein interaction (PPI) network construction and examination

The STRING database (http://string-db.org) (version 11.5) was used to construct the PPI network, which was visualized using Cytoscape software (version 3.9.1). The top 10 hub genes were identified using the cytoHubba plug-in in Cytoscape [18].

### Examining interactions of pathogen, lung microbiome, and host responses and correlating them with disease severity and outcomes

To construct microbiome-transcriptome network, we used spearman correlation to assess associations between the 76 DES and 1293 DEGs. Medium above correlations (*P* < 0 0.05 and |r| > 0.4) were visualized as an interactive network using Cytoscape version 3.9.1.

The linkET R package was used to calculate and visualize the associations among the clinical features, microbiome, and hub genes. Except for the correlation involving binary data using point-biserial correlation, all other data used Spearman correlation.

The patients were followed up weekly after discharge, and chest radiographs were reviewed according to the situation. Median values were used as cut-off values for continuous variables grouping studies. The follow-up time was defined from the date of BALF sampling to the last chest imaging assessment. The time to imaging recovery was defined as the time from BALF sampling to the apparent disappearance of large infiltrates on chest radiographs.

### Statistical analysis

Quantitatively normal or nearly normal data were expressed as the mean ± standard deviation and analyzed using the t-test. Quantitative skewness data were presented as the median (percentile: P25, P75) and analyzed using the Wilcoxon–Mann–Whitney rank-sum test. Categorical data are expressed as frequencies and examined using the chi-square test. Receiver operating characteristic curves (ROCs) and the area under the curve (AUC) were used to assess the accuracy of the diagnostic model. Log-rank tests were used for the Kaplan–Meier curves of the imaging recovery time. All statistical tests were two-sided, and *P* < 0.05 was considered statistically significant.

## Results

### Clinical characteristics

This study included 41 patients with MPP; twenty with various complications, including parapneumonic effusion, necrotic pneumonia, and respiratory failure, were assigned to the CMPP group (*n* = 20), whereas the others were assigned to the GMPP group (*n* = 21). The clinical and laboratory data of the patients with GMPP and CMPP are presented in Table 1. The median age of the patients with MPP was 7.0 years, and 25 (61.0%) were males. The levels of N%, CRP, ALT, LDH, PT, APTT, D-dimer, total fever duration, length of hospital stay, and hospital expenses were higher in the CMPP group than in the GMPP group (all *P* < 0.05). No significant differences were observed between the two groups in terms of age or sex distribution (*P* = 0.450 and 0.072, respectively).

**Table 1:** Demographics and clinical characteristics

Characteristics	Total (*N* = 41)	GMPP (*N* = 21)	CMPP (*N* = 20)	P
Sex, n (%)				
Male	25 (61.0)	10 (47.6)	15 (75.0)	0.072
Female	16 (39.0)	11 (52.4)	5 (25.0)	
Age, years	7.0 ± 2.8	6.6 ± 2.9	7.3 ± 2.7	0.450
WBC(×10^9^/L)	9.7 ± 4.1	9.3 ± 3.5	10.1 ± 4.6	0.518
N%	66.8 ± 11.9	60.2 ± 11.7	73.8 ± 7.5	< 0.001
Hemoglobin (g/L)	125.3 ± 11.2	126.0 ± 11.8	124.6 ± 10.8	0.705
Platelets (×10^9^/L)	292.3 ± 115.5	305.1 ± 144.7	278.9 ± 75.4	0.475
CRP (mg/L)	10.0 (2.8–29.5)	2.8 (2.8–12)	15.0 (4.37–35.5)	0.013
LDH (U/L)	419.0 (301.3–604.5)	313.5 (269.8–412.5)	547.0 (420.0–684.0)	0.001
ALT (U/L)	18.0 (12.3–26.0)	15.5 (10.5–18.8)	20.0 (15.0–38.3)	0.025
AST (U/L)	32.5 (24.5–44.0)	29.5 (23.3–36.0)	40.0 (26.5–49.3)	0.091
CK-MB (U/L)	20.0 (16.3–25.8)	20.0 (16.3–28.3)	20.0 (16.5–24.8)	0.892
PT (s)	12.8 (11.9–13.9)	12.1 (11.5–12.8)	13.5 (12.6–14.2)	0.001
APTT (s)	31.3 (27.4–34.5)	33.1 (29.7–34.9)	28.2 (25.7–32.7)	0.030
D-dimer (ng/ml)	564.5 (163.5–1870.5)	185.5 (116.0–548.8)	1827.0 (635.8–2686.0)	< 0.001
Fibrinogen (g/L)	3.7 (3.3–4.0)	3.6 (3.3–3.7)	3.7 (3.4–4.0)	0.171
Parapneumonic effusion, n (%)	18 (43.9)	0	18 (90.0)	< 0.001
Necrotizing pneumonia, n (%)	3 (7.3)	0	3 (15.0)	0.107
Respiratory failure, n (%)	3 (7.3)	0	3 (15.0)	0.107
Total fever duration (d)	9.0 (7.0–11.0)	8.0 (7.0–10.0)	10.0 (9.0–12.0)	0.013
Length of hospital stays (d)	10 (8.0–14.0)	9.0 (7.0 − 12.0)	12.5 (9.0–14.0)	0.032
Hospitalization expenses (CNY)	13,365.8 (11,714.9–17,107.33)	11,916.4 (10,719.3–13,288.6)	15,893.6 (14,231.0–25,034.4)	0.001
Duration of disease at sampling (d)	15.0 (13.0–18.0)	16.0 (13.0–19.0)	14.0 (13.0–17.0)	0.206

Quantitative normal or nearly normal data are expressed as the mean ± standard deviation. Quantitative skewness data are presented as the median (percentile: P25, P75), and categorical data are expressed as the frequency

*ALT* alanine transaminase, *AST* aspartate transaminase, *APTT* activated partial thrombin time, *CRP* C-reactive protein, *CMPP* complicated *Mycoplasma pneumoniae* pneumonia, *GMPP* general *Mycoplasma pneumoniae* pneumonia, *LDH* lactate dehydrogenase, *N%* neutrophil percentage, *PT* prothrombin time, *WBC* white blood cell

### Higher pathogen load in the CMPP group compared to the GMPP

**Fig. 1:**
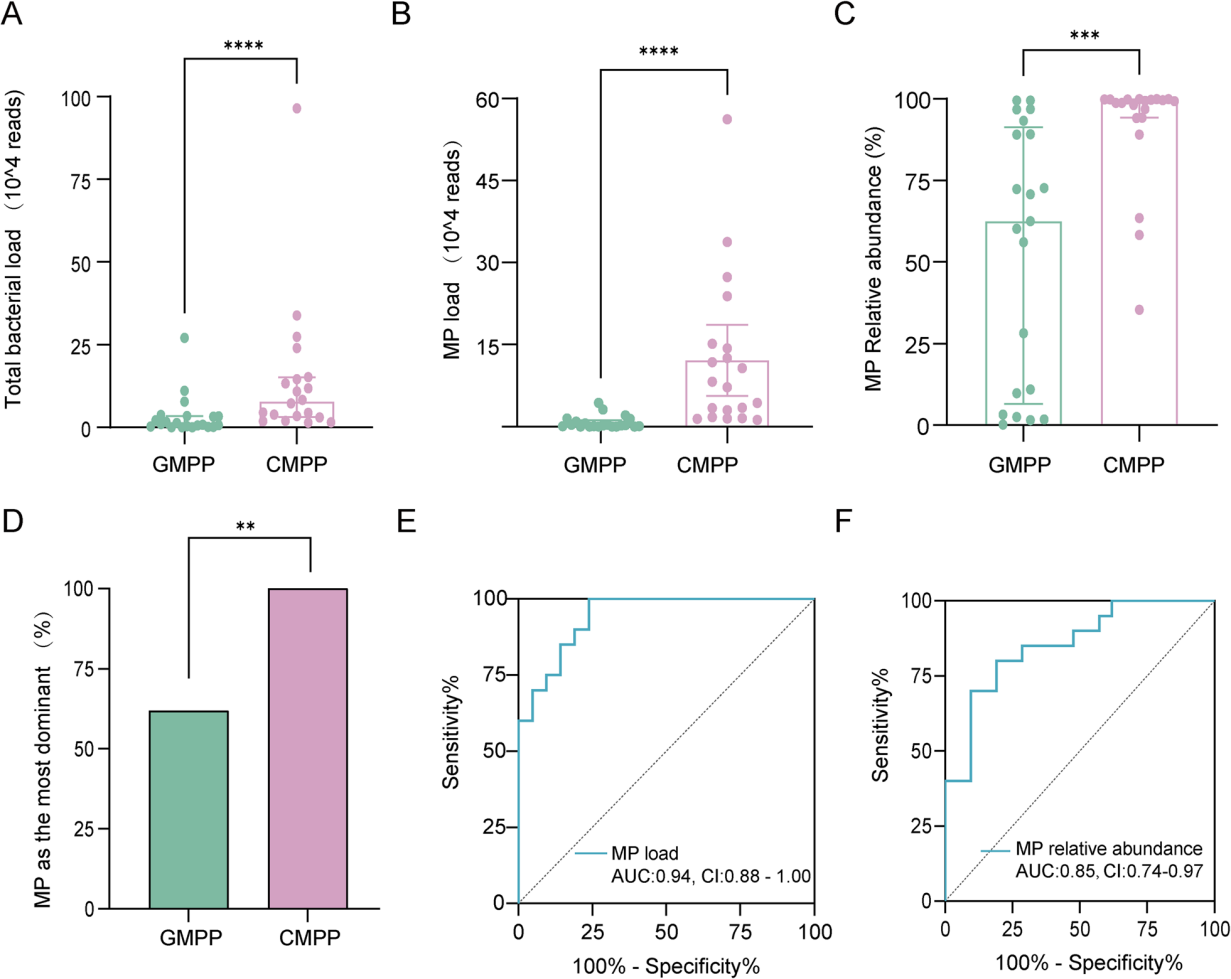
Pathogen load is associated with MPP severity. **A** The bacterial load in the CMPP and GMPP groups. **B** The MP load in the CMPP and GMPP groups. **C** The relative abundance of MP in the CMPP and GMPP groups. **D** The proportion of MP as the most dominant bacteria in CMPP and GMPP groups. **E** The diagnostic value of MP load for CMPP. **F** ROC curve constructed to study the diagnostic value of MP relative abundance for CMPP. *AUC* area under the curve, *CMPP* complicated *Mycoplasma pneumoniae* pneumonia, *CI* 95% confidence interval, *GMPP* general *Mycoplasma pneumoniae* pneumonia, *MP* *Mycoplasma pneumoniae*, *MPP* *Mycoplasma pneumoniae* pneumonia, *ROC* Receiver operating characteristic curve. *P < 0.05, **P < 0.01, ***P < 0.001, ****P < 0.0001

A higher bacterial load was found in the CMPP than in the GMPP (median, 7.7 vs. 1.0 104 reads, *P* = 0.0003) (Fig. 1A). The MP load and relative abundance were also higher in the CMPP group (median, 77,333 vs. 2,357 reads; *P* < 0.0001 and 99.07% vs. 62.50%, *P* < 0.0001, respectively) (Fig. 1B, C). Based on the percentage of species in each bacterial taxon, 33 (80.5%) of the 41 patients were identified as having a microbiome in which MP was the most abundant. The proportion of MP, the most dominant bacterium, was much higher in the CMPP group than in the GMPP group (100% vs. 69.1%, *P* = 0.002) (Fig. 1D). The MP load and relative abundance had significant diagnostic value for CMPP (Fig. 1E, F).

### Association of lung microbiota imbalance with MPP severity

**Fig. 2:**
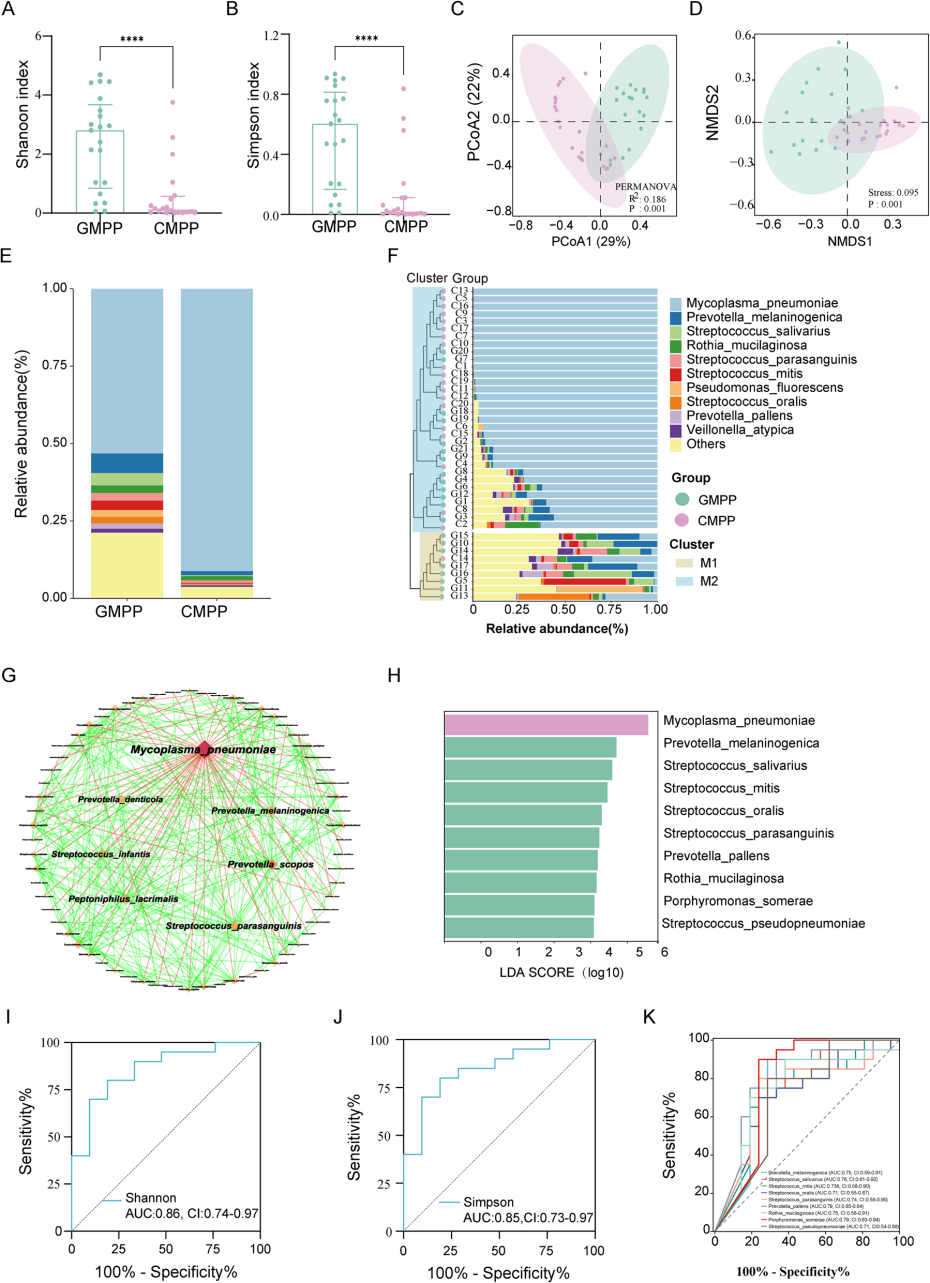
Lung microbiome imbalance are associated with MPP severity.  **A** Alpha diversity evaluated by the Shannon index. **B** Alpha diversity evaluated by the Simpson index. **C**, **D** Beta diversity evaluated by the PCoA and NMDS analyses based on Bray-Curtis distance. **E** Relative abundance of lung microbiota at the species level. **F** Hierarchical clustering-based classification of samples into two clusters, M1 and M2. **G** Network of associations among the lung microbiota. Diamond nodes represented species. The larger and redder the node, the more nodes it was associated with. Edges indicated significant associations ( P  < 0.05) between species and were colored based on positive (green) and negative (red) associations between species abundances. **H** The top 10 DES between the CMPP and GMPP groups based on LEfSe analysis. **I**-**J** The diagnostic value of alpha diversity for GMPP. (K) ROC curve constructed to study the diagnostic value of the top 10 DES (except MP) for GMPP.  *AUC* area under the curve, *CI* 95% confidence interval, *DES* differentially expressed species, *LEfSe* linear discriminant analysis (LDA) effect size, *NMDS* non-metric multidimensional scaling, *PCoA* principal coordinates analysis, *ROC* receiver operating characteristic curve

NGS revealed the differences in the lung bacterial communities in the CMPP and GMPP groups. No significant differences were observed between the two groups in the richness estimator, abundance-based coverage estimator (ACE), and Chao1 estimator (*P* = 0.12 and *P* = 0.27, respectively) (Additional file 1: Fig. S1A, B). However, the alpha diversity, as determined by the Shannon and Simpson indices, indicated a significant decrease in the CMPP group compared to the GMPP group (*P* = 0.0003 and *P* = 0.0007, respectively) (Fig. 2A, B). Beta diversity analysis of PCoA and NMDS revealed that the lung microbiota from the different groups were largely separated (Fig. 2C, D).

MP was the dominant species in the lung microbiome of patients with MPP, followed by *Prevotella melaninogenica, Streptococcus salivarius, Rothia mucilaginosa, Streptococcus parasanguinis*, and *Streptococcus mitis* (Fig. 2E). Hierarchical clustering analysis based on the Bray weighting method classified the samples into two clusters, M1 (*n* = 9) and M2 (*n* = 32) (Fig. 2F). The CMPP percentage in the M2 cluster was higher compared to the M1 cluster (59.4% vs. 0; *P* = 0.002). The network of associations among lung microbiome showed that MP exhibited the highest number of associations with other species and the associations were all negative.

Top 10 species of the 76 DES examined through LEfSe are shown in Fig. 2H. The Shannon and Simpson indices showed good predictive values for GMPP (AUC = 0.860, *P* < 0.0001 and AUC = 0.850, *P* = 0.0001, respectively; Fig. 2I, J). The relative abundance of DES (except for MP) also had a significant diagnostic value for GMPP (Fig. 2K).

### Correlation of CMPP host transcriptional features with neutrophils functions and inflammatory response pathways

#### DEGs and associated pathways

**Fig. 3:**
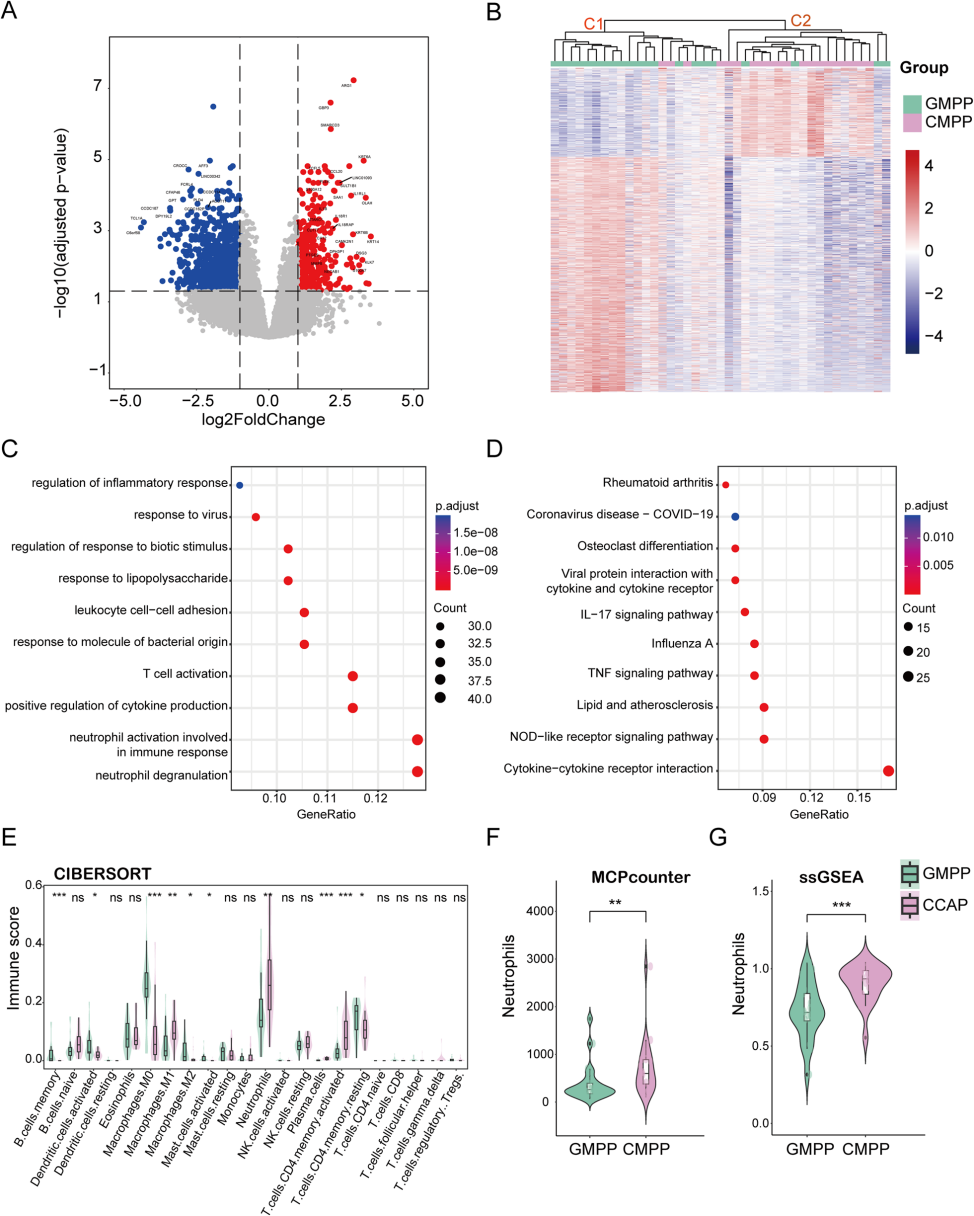
Host transcriptional features of CMPP are correlated with neutrophil functions and inflammatory response pathways.  **A** Volcano plot of DEGs in CMPP and GMPP. The horizontal line at adj P  = 0.05; vertical line at |log 2 FC| = 1. Red and blue dots in volcano plot show upregulated and downregulated genes, respectively. **B** Heatmap showing the DEGs. The gradation of color represents the value of |lo g2 FC|. Hierarchical clustering classified samples into two clusters, namely the C1 cluster and the C2 cluster. **C**, **D** GO-Biological Process, KEGG enrichment analysis of the up regulated DEGs. **E** A violin plot showing the distribution of 22 types of immune cells in CMPP and GMPP. **F**, **G** Comparison of the infiltration of neutrophils between the two groups using other algorithms.  *BP* biological process, *DEG* differentially expressed gene, *DEC* differentially expressed cell, *FC* fold change, *GO* Gene Ontology, *KEGG* Kyoto Encyclopedia of Genes and Genomes, *ssGSEA* single-sample gene set enrichment analysis

Between CMPP and GMPP, 1,293 DEGs were detected, of which 355 were upregulated and 938 were downregulated (Fig. 3A, B). Hierarchical clustering classified the samples into two clusters: C1 (*n* = 21) and C2 (*n* = 20) (Fig. 3B). The CMPP percentage in the C2 cluster was higher than that in the C1 cluster (81.0% vs. 19.1%; *P* < 0.0001). GO enrichment analysis indicated that the upregulated DEGs were mainly concentrated in neutrophil degranulation and positive regulation of cytokine production (Fig. 3C). The KEGG pathways of the upregulated DEGs were mainly enriched in the cytokine-cytokine receptor interaction, NOD-like receptor signaling pathway, and IL-17 signaling pathway (Fig. 3D).

### Immune cells landscape

The differences in the distribution of 22 types of immune cells between the two groups are shown in Fig. 3E, with ten differentially expressed cells (DECs) identified: plasma cells (*P* < 0.001), memory activated CD4 T cells (*P* = 0.001), M1 Macrophages (*P* = 0.006), and Neutrophils (*P* = 0.007) were upregulated, whereas memory B cells (*P* < 0.001), M0 Macrophages (*P* < 0.001), and activated mast (*P* = 0.030), and dendritic (*P* = 0.002) cells were downregulated in the CMPP group. Using other algorithms, we confirmed the significantly higher abundance of neutrophils in the CMPP group compared to the GMPP group (Fig. 3F, G).

### CMPP-related module genes

**Fig. 4:**
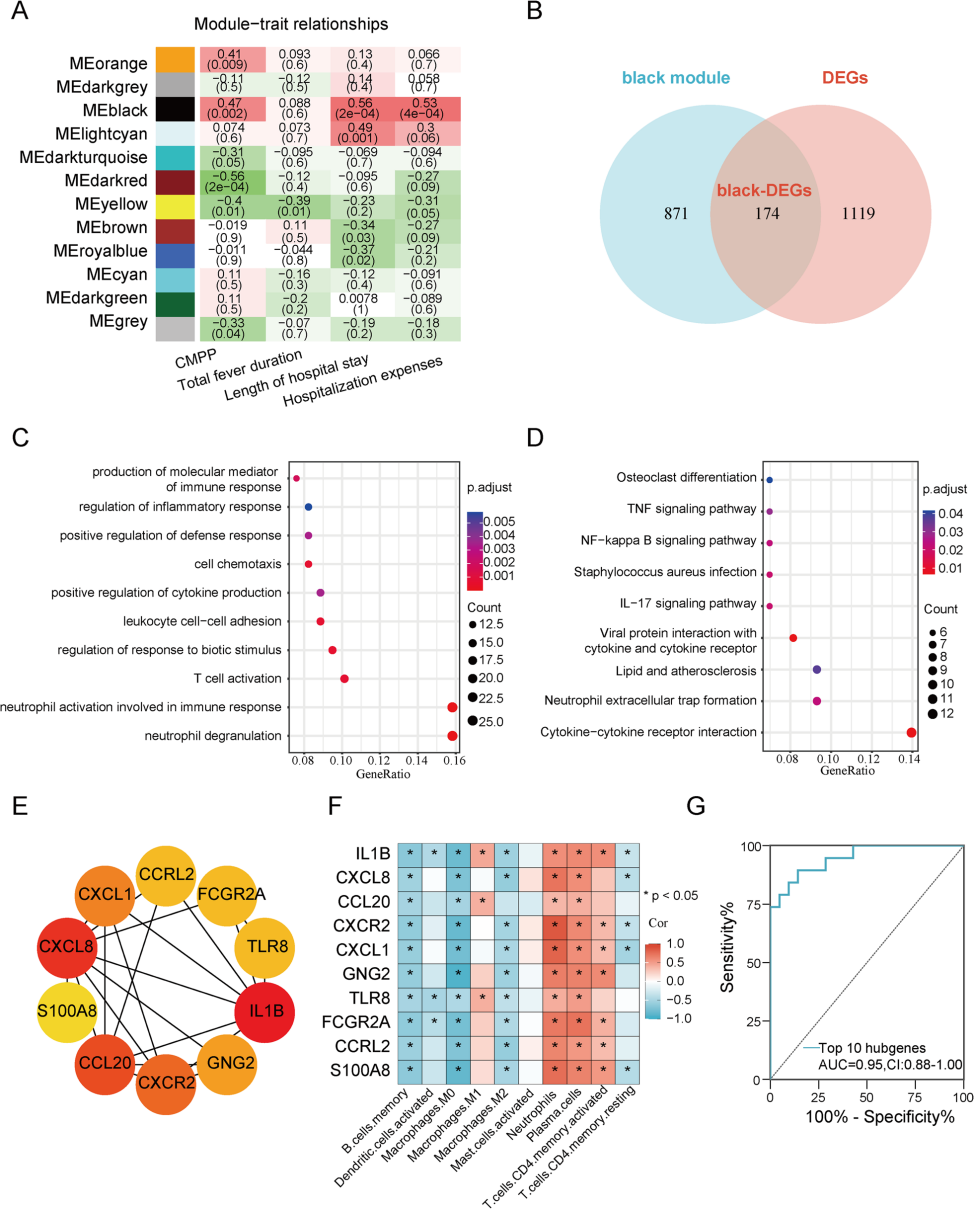
CMPP-related module genes are correlated with neutrophil functions and inflammatory response pathways.  **A** The WGCNA used to analyze all the genes and identify the modules significantly related to traits. Heatmaps show the correlation between eigengenes and clinical traits. The cells are colored by the correlation according to the color legend. Each row corresponds to a module eigengene. Each cell contains the corresponding correlation and P value. **B** The Venn diagram displaying the black-DEGs overlapping in the black module and DEGs between CMPP and GMPP. **C**, **D** GO and KEGG enrichment of the black-DEGs genes. **E** CytoHubba-MCC was used to identify the top 10 hub genes in the network. The darker the orange color, the higher the score. **F** The relationship between DECs and hub black DEGs. **G** ROC curve constructed to study the predictive effect of the 10 hub genes on the diagnosis of CMPP.  *AUC* area under the curve, *CI* 95% confidence interval, *ROC* receiver operating characteristic curve, *WGCNA* weighted Gene Co-expression Network analysis. *P  < 0.05, ** P  < 0.01, *** P  < 0.001, **** P  < 0.0001

WGCNA, performed using the expression profiles of all genes to identify the co-expression modules related to CMPP, with a soft-threshold of 10 resulting in a scale-free network (Additional file 1: Fig. S2A, B), divided all genes into 12 modules, each with a unique color (Additional file 1: Fig. S2C). Among the 12 modules, the dark module was highly associated with clinical data (CMPP, length of hospital stay, and hospitalization expenses) (Fig. 4A). By integrating 1045 genes in the black module with the 1293 DEGs between CMPP and GMPP, 174 DEGs (black) were obtained (Fig. 4B) and selected as clinically important modules for further analysis.

### Functional enrichment analysis of key module

GO enrichment of the black-DEGs showed that they were mainly concentrated in neutrophil degranulation and neutrophil activation involved in immune response (Fig. 4C). KEGG pathways enrichment indicated their association with cytokine-cytokine receptor interaction, neutrophil extracellular trap formation, and cytokine receptor, IL-17 signaling pathway (Fig. 4D).

### PPI network and hub genes

The PPI network for the 174 black DEGs constructed and visualized using the STRING database and Cytoscape software, respectively, is given in Fig. S3. The top 10 hub genes in the PPI network determined by cytoHubba were *IL1B, CXCL8, CCL20, CXCR2, CXCL1, GNG2, TLR8, FCGR2A, CCRL2*, and *S100A8* (Fig. 4E), all of which were upregulated in the CMPP group.

Furthermore, the 10 hub genes were positively correlated with the most upregulated DECs, whereas they were negatively correlated with most downregulated DECs (Fig. 4F). In particular, the CIBERSORT immune score of neutrophils was positively correlated with all hub black DEGs.

The predictive effect of the joint indicator of the top 10 hub genes in the diagnosis of CMPP was observed through a ROC curve. Figure 4G shows that the hub genes had high diagnostic ability for CMPP (AUC = 0.95; *P* < 0.001).

### Association of interactions among pathogens, lung microbiome, and host response with disease severity and outcomes

**Fig. 5:**
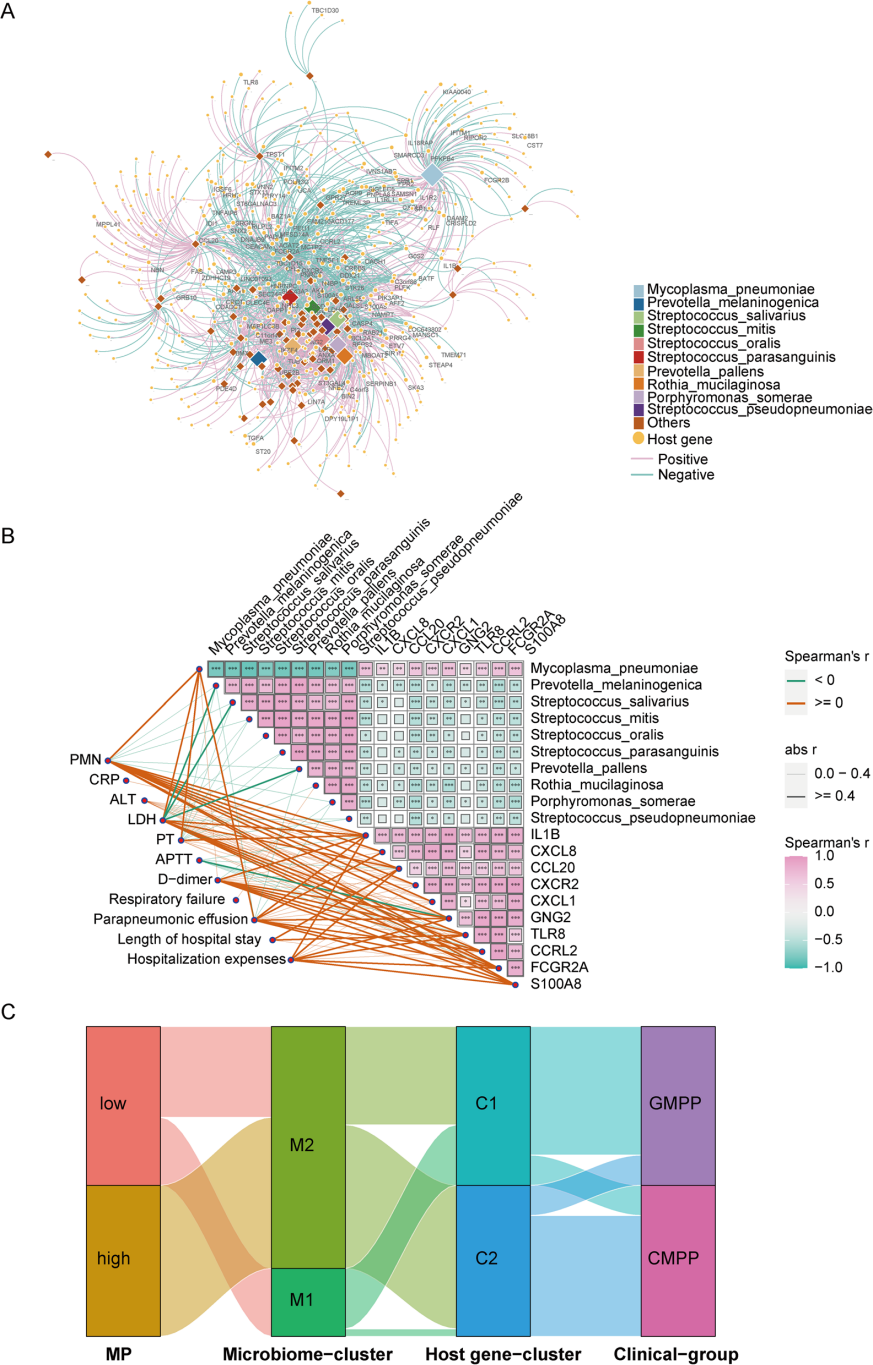
Interactions among pathogens, lung microbiome, and host responses are associated with disease severity.  **A** Network of associations between the lung DES (n  = 76) and DEGs (n  = 1293) between CMPP and GMPP groups. Each diamond node represented a lung species. Each circle node represented a host gene. Edges showed medium above associations between microbial abundance and gene expression level (Spearman’s r  > 0.4, P  < 0.05). The circular nodes labeled were black-DEGs. **B** A heatmap showing the correlation among lung microbiome (top 10 DES), host response (top 10 hub genes) and clinical indicators. **C** Sankey diagram showing the relationships among pathogen, lung microbiome, host response, and clinical features

To characterize associations between lung microbiome and the transcriptome in children with MPP, we constructed association network between DES and DEGs (Fig. 5A). The mean number of DEGs with each lung species in this network was 128.8. MP interacted with 344 DEGs, 100 of which were black-DEGs, representing 57.5% of the total number of black-DEGs.

A correlation matrix analysis of MP load, lung microbiome indicators (top 10 DES, except for MP), host response indicators (the expression of top 10 hub black DEGs), and clinical indicators, including N%, CRP, ALT, LDH, PT, APTT, and D-dimer, indicated the potential interactions among the three core elements of respiratory infection: pathogen, lung microbiome, and host response. Figure 5B shows that the interplay between MP, lung microbiome, and host response determines critical clinical outcomes.We classified these three core elements into modules. Patients were divided into two clinical groups (MP-high and MP-low) based on the median relative abundance of MP, two microbiome clusters (M1 and M2) depending on the lung microbiome clustering map, and two host gene clusters (C1 and C2) based on the host gene expression clustering map. A Sankey diagram was used to visualize the relationships among the three core elements and clinical features (Fig. 5C). The MP-high group mapped almost entirely to the microbiome M2, host gene cluster C2, and clinical module CMPP. Patients in the MP-high group manifested a specific lung microbiome structure and host response type and were more prone to developing CMPP.

**Fig. 6:**
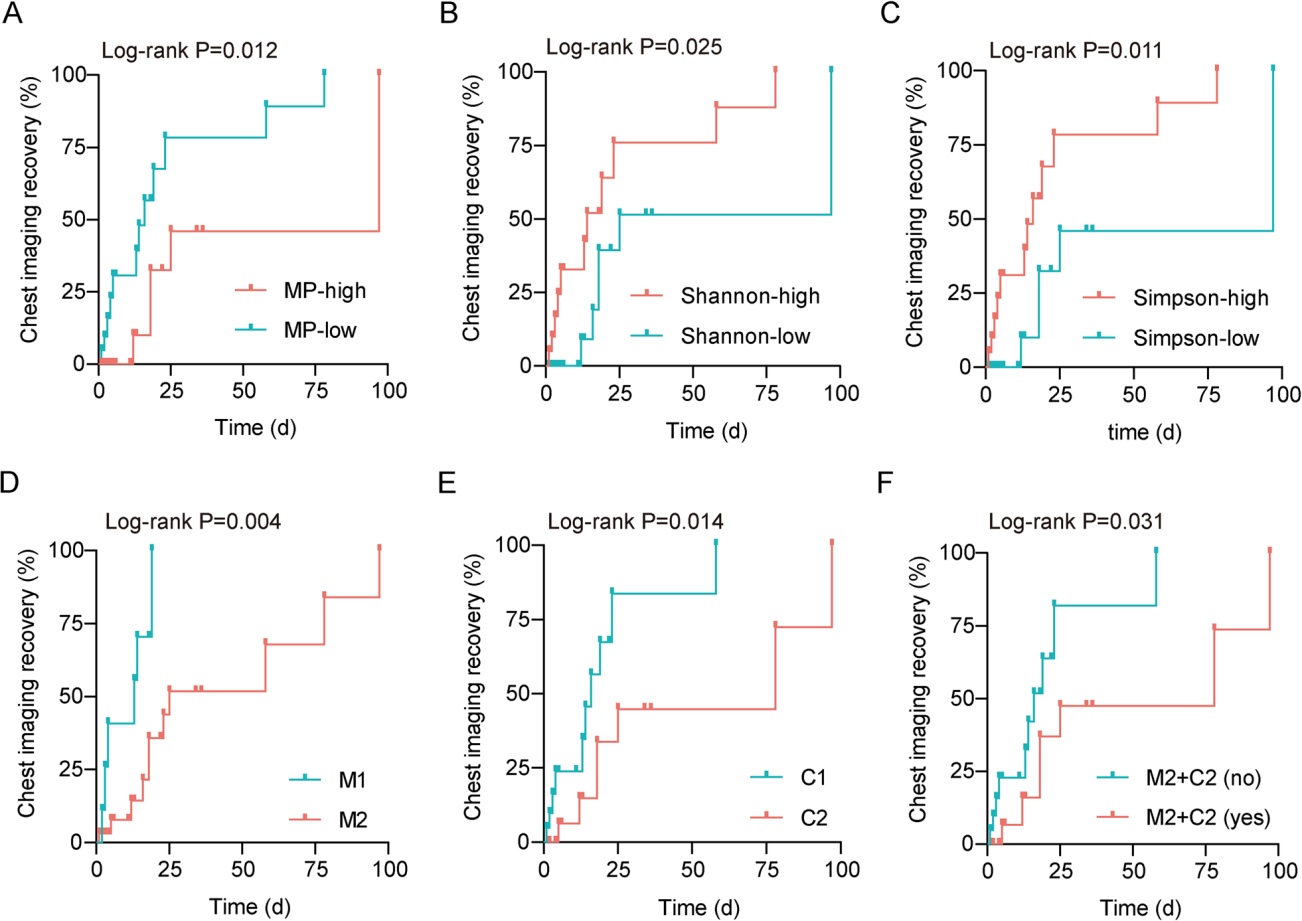
Interactions among pathogens, lung microbiome, and host responses are associated with outcomes.  Kaplan–Meier survival curves for chest imaging recovery based on the relative abundance of MP (**A**), Shannon index (**B**), Simpson index (**C**), microbiome cluster (**D**), host gene cluster (**E**), combination module of microbiome cluster M2, and host gene cluster C2 (**F**)

Ultimately, the imaging recovery time of children with MPP was estimated using a Kaplan–Meier survival curve. The MP load, lung microbiome (Shannon and Simpson indices and microbiome clusters), and host gene clusters were used as variables for survival analysis (Fig. 6A–F). High relative abundance of MP (*P* = 0.012), low alpha diversity (Shannon: *P* = 0.025; Simpson: *P* = 0.011, respectively), microbiome cluster M2 (*P* = 0.004), host gene cluster C2 (*P* = 0.014), and the combination module of M2 and C2 (*P* = 0.031) were associated with long imaging recovery time.

## Discussion

CMPP poses a challenge for the treatment of patients with MPP. It is associated with excessive host immunity activation, and studies have shown an essential role of the airway microbiome [14, 15, 21]. Therefore, a cohort study was conducted using BALF samples and integrated multi-omics analyses to investigate the interactions among pathogen, lung microbiome, and host response, and their relevance to severity and outcomes in children with MPP.

MP was the most abundant species in the lung microbiome of 80.5% of patients with MPP and 100% of patients with CMPP. MP occupied almost all niches in most patients with CMPP, with the abundance of other species being very low [14, 21]. Children with MPP with mucus plugs had a higher abundance of Mycoplasma compared with those without mucus plugs [14]. Moreover, Chen et al. argued that MP-dominated microbial communities were a characteristic of the lower respiratory tract microbiome of children with refractory MPP [21].

Previous studies have demonstrated an unbalanced airway microbiome in patients with MPP, whether in the upper airway, represented by nasopharynx and oropharynx swabs, or in the lower airway, represented by BALF samples [9, 22, 23]. MP directly competes with colonized bacterial commensals in the lungs, resulting in a simpler co-occurrence network in patients with MPP compared to healthy children [22, 23]. The indigenous microbiota itself plays an essential role in excluding pathogenic expansion by modulating host responses to maintain homeostasis [10]. Disruptions of the airway microbiota can result in altered disease severity and outcomes.

Between the CMPP and GMPP groups, 87 DES were recovered. They were downregulated in the GMPP group and had a significant diagnostic value for GMPP, except for MP. Most of these were oral commensal bacteria, such as *P. melaninogenica, S. salivarius, S. mitis, S. oralis*, and *S. parasanguinis*, which may function as pathogen competitors. They have constant access to the lower airways via micro aspiration [24]. *Prevotella* is consistently the core bacteria of the respiratory tract [25, 26]. Previous studies have demonstrated that commensal bacteria can interact with airway pathogens, such as *Pseudomonas aeruginosa*, to improve lung function and clinical stability [27, 28]. As a result, we assumed that the specific airway microbiome composed of these commensal bacteria could inhibit MP and maintain lung environmental homeostasis.

The etiology and pathogenesis of CMPP remain unclear, and excessive host immune responses have been reported to play an important role. Our previous work has confirmed that peripheral blood neutrophils levels are significantly increased in children with refractory MPP [1, 29, 30]. In this study, host gene analysis revealed a key module associated with neutrophil functions and many inflammatory response pathways, such as cytokine-cytokine receptor interaction, neutrophil extracellular trap formation, and the IL-17 signaling pathway. Additionally, we observed increased neutrophils levels in the CMPP group, both in clinical blood sample tests and the deconvolution of bulk RNA sequencing of immune cells in the BALF samples. Transcriptome sequencing of neutrophils from the peripheral blood of children with MPP further confirmed the role of neutrophils, particularly neutrophil extracellular trap formation, in MPP [16]. Cytokines play critical roles in the pathogenesis of MPP, and cytokine storms are associated with MPP severity [31, 32]. Most hub genes in the key module that expressed cytokines or chemokines and their receptors, such as *IL1B, CXCL8, CCL20, CXCR2, CXCL1*, and *CCRL2*, were increased in CMPP, and had a high predictive value for CMPP. Given that IL-17 induces the secretion of chemokines to recruit neutrophils for host defense, producing antimicrobial peptides to maintain lung barrier function, it can be protective; however, excessive IL-17 may result in persistent neutrophil recruitment, degranulation, and tissue damage; however, excessive IL-17 may result in persistent neutrophil recruitment, degranulation, and tissue damage [33, 34]. Previous studies have reported the association of IL-17 with MPP progression and outcomes, including MP-associated asthma and bronchiolitis obliterans [35–37]. Therefore, neutrophil function, cytokine storms and IL-17 signaling may become novel targets for treatment of MPP treatment.

The lung microbiota is essential in regulating pulmonary immune responses; however, their role in host-specific immunity is yet to be evaluated comprehensively. Using combined multi-omics analysis, we found that the MP relative abundance was negatively correlated with other lung microflora and diversity indices and positively correlated with hub gene expression and clinical indicators of inflammation and organ damage. Patients in the MP-high relative abundance group, manifested by a specific microecological structure and host immune response type, were more likely to develop CMPP and had a long pneumonia recovery time. Nevertheless, whether airway microbial alterations are the cause or consequence of the host immune response requires further investigation. Dickson et al. proposed a comprehensive model: respiratory tract dysbiosis provokes a dysregulated host immune response, altering the growth conditions for microbes in the airways, promoting further dysbiosis and perpetuating a cycle of inflammation and disordered microbiota [38]. As a result, any interruption in the treatment regimen aimed at disrupting this vicious cycle may alleviate the disease severity and improve the outcomes of patients with MPP.

However, the number of cases incorporated into this study was relatively small. Therefore, further large-size, prospective studies are required to provide advanced evidence for the interactions among the pathogen, lung microbiome, and host response in MPP.

## Conclusion

We performed a cohort study and multi-omics integration analysis of BALF in 41 patients with MP to correlate pathogen, lung microbiome, and host response, and associate the findings with observed clinical features and outcomes. Our findings indicate that MP, the lung microbiome, and the host immune response interact with each other and are closely related to disease severity and outcomes in children with MPP.

### Supplementary information

**Additional file 1: Fig. S1.**  Comparisons of the lung microbiome in the BALF from CMPP and GMPP groups. **Fig. S2.**  The WGCNA method was used to analyze all the genes and to find out the modules significantly related to traits. **Fig. S3.**  The PPI network of black-DEGs.

## Data Availability

The raw sequence data reported in this paper have been deposited in the Genome Sequence Archive (Genomics, Proteomics & Bioinformatics 2021) in National Genomics Data Center (Nucleic Acids Res 2022), China National Center for Bioinformation / Beijing Institute of Genomics, Chinese Academy of Sciences (GSA-Human: HRA004226) that are publicly accessible at https://ngdc.cncb.ac.cn/gsa-human.
