# Supplementary material for: Integrative study of pulmonary microbiome, transcriptome and clinical outcomes in Mycoplasma pneumoniae pneumonia
Source: Respir Res. 2024 Jan 18;25:35. doi: 10.1186/s12931-024-02687-4 (PMC10795342; doi:10.1186/s12931-024-02687-4)
Supplement: Supplementary file 1 — Additional file 1: Fig. S1. Comparisons of the lung microbiome in the BALF from CMPP and GMPP groups. Fig. S2. The WGCNA method was used to analyze all the genes and to find out the modules significantly related to traits. Fig. S3. The PPI network of black-DEGs. [file 12931_2024_2687_MOESM1_ESM.docx]

Additional Materials for

**Integrative study of pulmonary microbiome, transcriptome and clinical outcomes in *Mycoplasma pneumoniae* pneumonia**


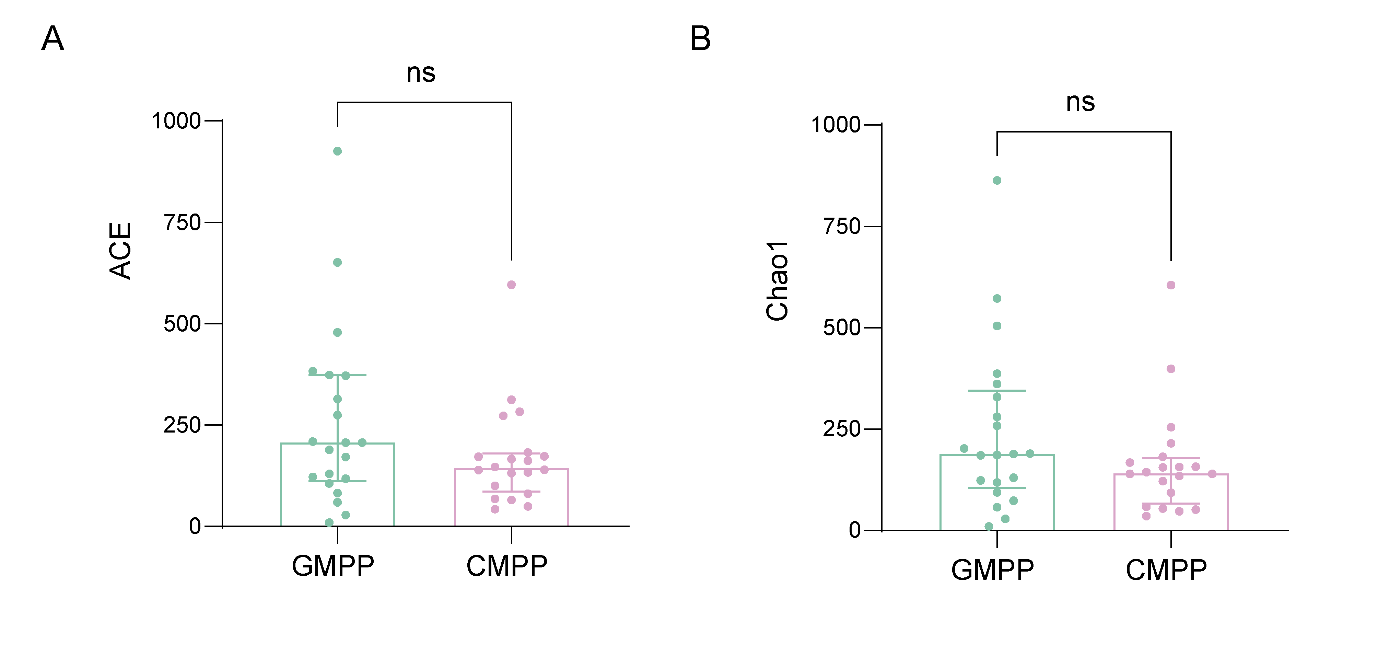


**Fig. S1 Comparisons of the lung microbiome in the BALF from CMPP and GMPP groups.**

(A-B) Richness was evaluated by the Chao1 and ACE.

ACE: Abundance-based coverage estimator; BALF: bronchoalveolar lavage fluid; CMPP: Complicated *Mycoplasma pneumoniae* pneumonia; GMPP: General *Mycoplasma pneumoniae* pneumonia.


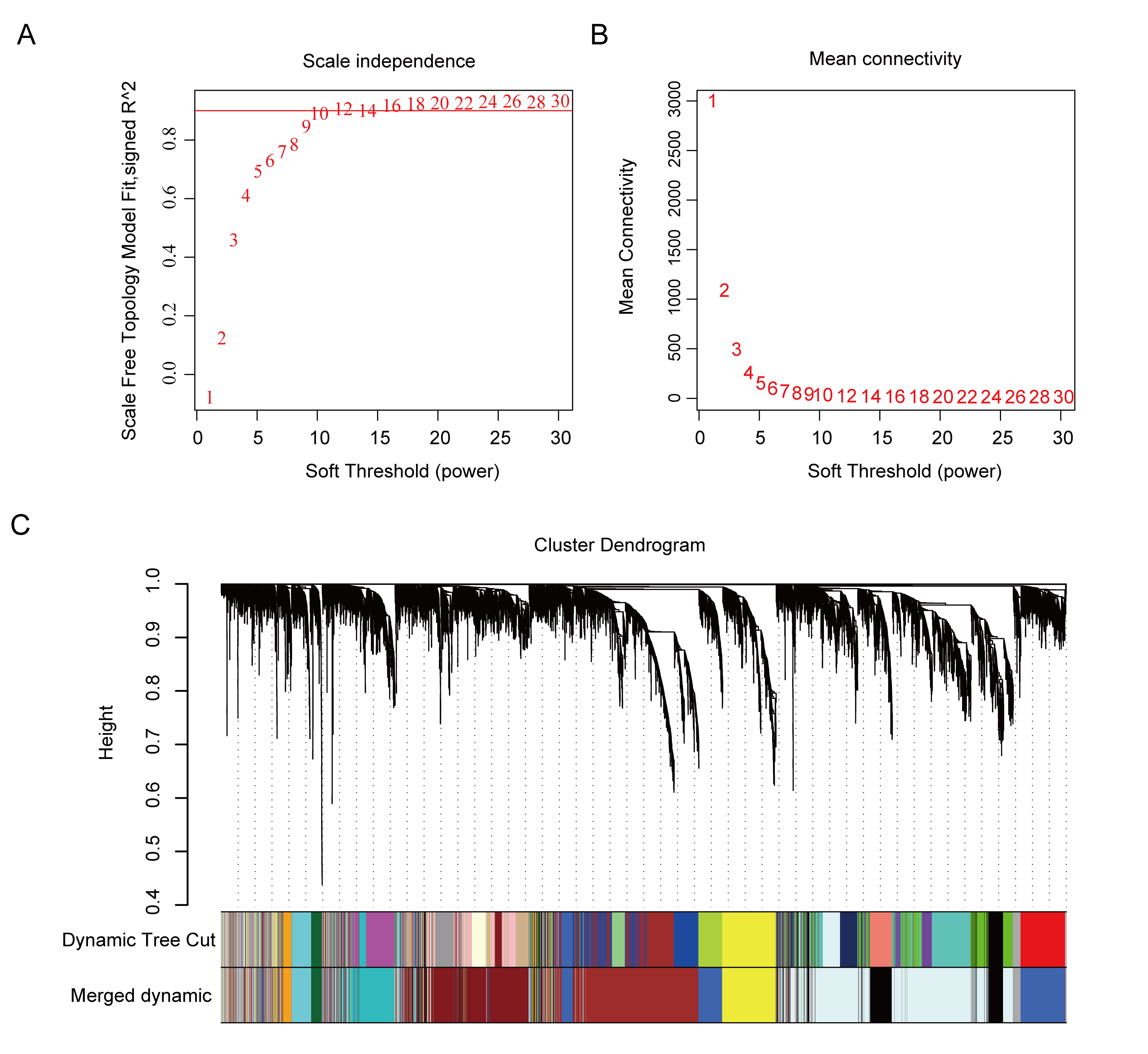


**Fig. S2 The WGCNA method was used to analyze all the genes and to find out the modules significantly related to traits.**

(A) The relationship between the scale-free fit index and various soft-thresholding powers; (B) The relationship between the mean connectivity and various soft-thresholding powers; (C) Clustering dendrogram of genes, various colors represent different modules.

WGCNA: Weighted Gene Co-expression Network Analysis.


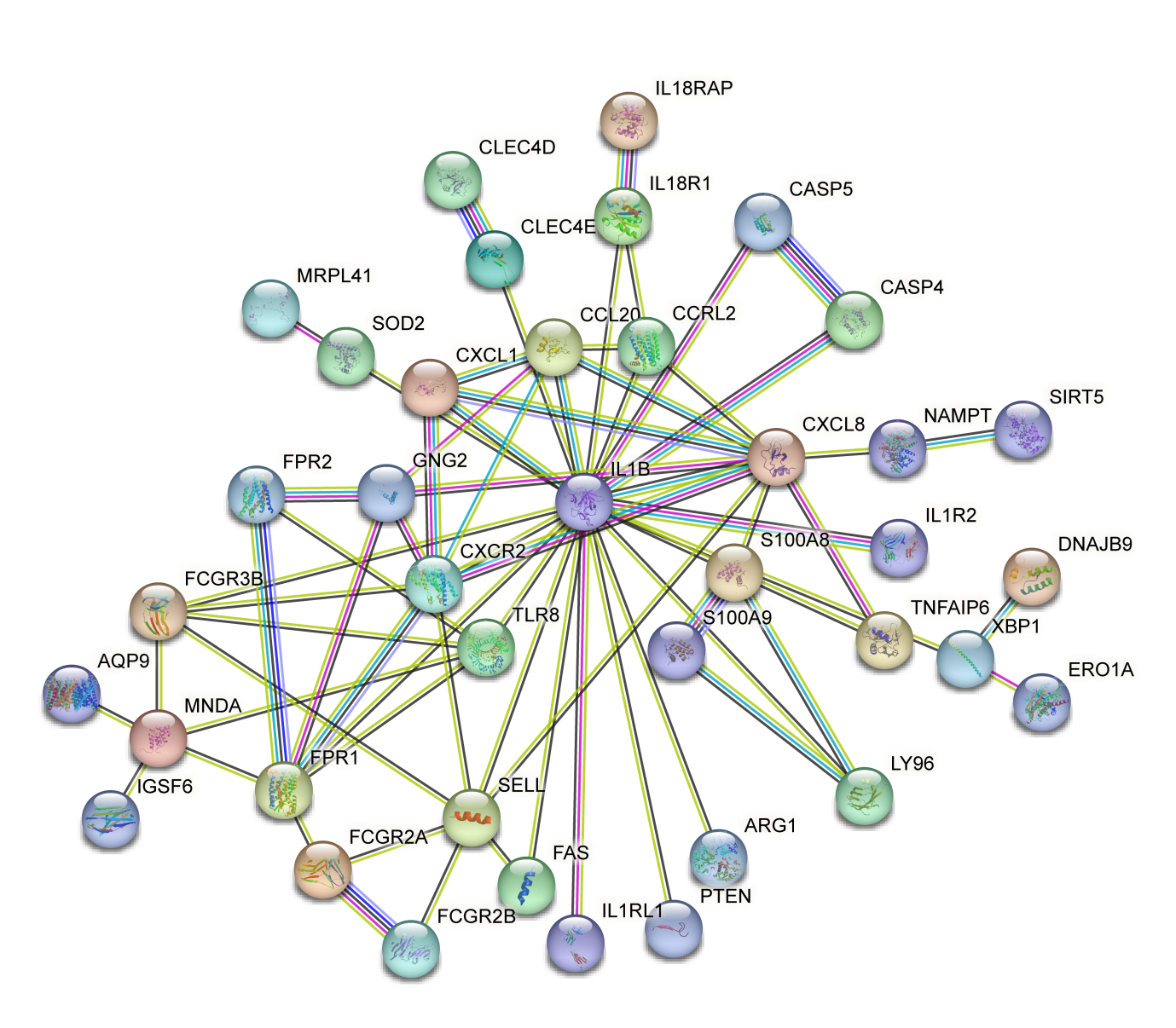


**Fig. S3 The PPI network of black-DEGs.**

Differentially expressed gene; PPI: protein-protein interactions.
